# Duration of participation in continuous quality improvement: a key factor explaining improved delivery of Type 2 diabetes services

**DOI:** 10.1186/s12913-014-0578-1

**Published:** 2014-11-19

**Authors:** Veronica Matthews, Gill Schierhout, James McBroom, Christine Connors, Catherine Kennedy, Ru Kwedza, Sarah Larkins, Elizabeth Moore, Sandra Thompson, David Scrimgeour, Ross Bailie

**Affiliations:** Menzies School of Health Research, Brisbane, Queensland Australia; School of Environment, Griffith University, Brisbane, Queensland Australia; Department of Health, Darwin, Northern Territory Australia; Maari Ma Health Aboriginal Corporation, Broken Hill, Far West New South Wales Australia; Queensland Health, Cairns, Queensland Australia; School of Medicine & Dentistry, James Cook University, Townsville, Queensland Australia; Aboriginal Medical Services Alliance Northern Territory, Alice Springs, Northern Territory Australia; Western Australian Centre for Rural Health, University of Western Australia, Geraldton, Western Australia Australia; Aboriginal Health Council of South Australia, Adelaide, South Australia Australia

**Keywords:** Quality improvement, Aboriginal & Torres Strait Islander populations, Type 2 diabetes mellitus, Primary health care, Variation in care

## Abstract

**Background:**

It is generally recognised that continuous quality improvement (CQI) programs support development of high quality primary health care systems. However, there is limited evidence demonstrating their system-wide effectiveness. We examined variation in quality of Type 2 diabetes service delivery in over 100 Aboriginal and Torres Strait Islander primary health care centres participating in a wide-scale CQI project over the past decade, and determined the influence of health centre and patient level factors on quality of care, with specific attention to health centre duration of participation in a CQI program.

**Methods:**

We analysed over 10,000 clinical audit records to assess quality of Type 2 diabetes care of patients in 132 Aboriginal and Torres Strait Islander community health centres in five states/territories participating in the ABCD project for varying periods between 2005 and 2012. Process indicators of quality of care for each patient were calculated by determining the proportion of recommended guideline scheduled services that were documented as delivered. Multilevel regression models were used to quantify the amount of variation in Type 2 diabetes service delivery attributable to health centre or patient level factors and to identify those factors associated with greater adherence to best practice guidelines.

**Results:**

Health centre factors that were independently associated with adherence to best practice guidelines included longer participation in the CQI program, remoteness of health centres, and regularity of client attendance. Significantly associated patient level variables included greater age, and number of co-morbidities and disease complications. Health centre factors explained 37% of the differences in level of service delivery between jurisdictions with patient factors explaining only a further 1%.

**Conclusions:**

At the health centre level, Type 2 diabetes service delivery could be improved through long term commitment to CQI, encouraging regular attendance (for example, through patient reminder systems) and improved recording and coordination of patient care in the complex service provider environments that are characteristic of non-remote areas.

## Background

The international diabetes epidemic is expected to escalate over coming decades placing increasing economic and social pressures on health systems and communities [1-3]. In addition, socio-economic and ethnic inequities have been identified in provision of diabetes care [4]. Social determinants and lack of access to quality primary health care (PHC) services are important contributors to the inequity in health outcomes between Aboriginal and Torres Strait Islander communities and the Australian non-Indigenous population. Over the past decade, there has been a significant increase in prevalence of diabetes in the Aboriginal and Torres Strait Islander population and they are now three times more likely to have diabetes compared to the non-Indigenous Australian population [5].

Along with targeted prevention strategies, improvements in chronic disease care are necessary to reduce inequity, advance population health outcomes and lessen the burden on health care systems. Evidence indicates that improvements in quality of care and health outcomes can be achieved using integrated care frameworks and multifaceted improvement strategies targeting changes at all levels of the health system [6-9]. However, the effectiveness of local and large scale quality improvement approaches in health care remains uncertain with published studies showing considerable variation in levels of improvements achieved [10,11].

### Australian Aboriginal and Torres Strait Islander primary health care setting

There are a number of service sectors providing primary health care to Aboriginal and Torres Strait Islander communities. In recognition of the need for culturally appropriate care, Aboriginal and Torres Strait Islander community-controlled health services were developed over 40 years ago, employing a comprehensive care model with local Aboriginal and Torres Strait Islander participation and control in guiding, planning and delivery of services. The success of the community-controlled sector has led other health care providers to adopt community participatory processes in health service design, including a State and Territory government operated community health centre predominantly servicing an Indigenous community [12]. Both of these sectors are represented in metropolitan, remote and very remote locations across Australia.

### One21seventy/ABCD CQI program

Over the past decade, a wide scale continuous quality improvement (CQI) program operating in both Aboriginal and Torres Strait Islander community controlled and government centres has included a focus on Type 2 diabetes. The Audit and Best Practice for Chronic Disease (ABCD) project employs a systems approach to improving PHC delivery utilising evidence-based clinical audit and system assessment tools to enable health centres to assess and reflect on system performance [13,14]. Typically, health centres conduct audits on an annual basis choosing from a suite of tools covering various aspects of PHC (including Type 2 diabetes, chronic kidney disease, coronary heart disease, maternal and child health, preventive health and mental health). A not-for-profit service agency, One21seventy, develops and maintains evidence-based audit and systems assessment tools, provides online data services for automated reporting, benchmarking and interpretation, and training and site support for conducting audits according to standard protocols. Over 100 health centres using the One21seventy/ABCD tools have voluntarily provided their de-identified audit data to the ABCD National Research Partnership for analysis of variation in PHC service delivery.

The aim of this study is to examine trends in the quality of Type 2 diabetes processes of care over time for participating health centres and identify the influence of regional, health centre, and individual patient level factors on delivery of services scheduled in current guidelines. Specifically, the analysis aimed to explore whether or not health centre duration of participation in the ABCD CQI program was associated with improvements in Type 2 diabetes service delivery.

## Methods

### Study context and design

Table 1 shows completion rates of Type 2 diabetes audits by year of commencement in the project. Coinciding with stepped phases of research and geographical extensions of the project, a large number of health centres joined the program in 2006 (n = 41) and 2011 (n = 40). Participation in annual Type 2 diabetes audits is driven by health centre decision processes based on local priorities. As such, not all health centres completed Type 2 diabetes audits in consecutive years and CQI activity may have focussed on other clinical areas in-between diabetes audit cycles. This retrospective longitudinal study used Type 2 diabetes audit data to examine changes in quality of care over time and explore factors underlying variation in care delivery.

**Table 1:** **Health centre participation in Type 2 diabetes audits by year of commencement**

**First year of participation**	**Completed baseline only**	**Completed 1–2 cycles**	**Completed ≥3 cycles**	**Total**
	**n (%)**	**n (%)**	**n (%)**	
2006	2 (5)	11 (27)	28 (68)	41
2007	2 (20)	3 (30)	5 (50)	10
2008	1 (11)	3 (33)	5 (56)	9
2009	0 (0)	2 (22)	7 (78)	9
2010	3 (25)	9 (75)		12
2011	13 (33)	27 (68)		40
2012	11 (100)			11
Total	32 (24)	55 (42)	45 (34)	132

### Data collection

Clinical audit records from 132 Aboriginal and Torres Strait Islander community health centres in five states/territories participating in the ABCD project between 2005 and 2012 were analysed to assess quality of Type 2 diabetes care of patients. Clinical audits are generally completed by health centre staff trained in the use of One21seventy/ABCD tools and supported by quality improvement facilitators and/or One21seventy staff. The Type 2 diabetes audit tool was developed by an expert working group, with participation of chronic illness experts and health service staff from a number of jurisdictions across Australia. The tool is designed to enable services to assess their actual practice against best practice standards, and is accompanied by a protocol that includes reference to the guidelines that form the basis of the tool. Inter-rater validation tests were carried out as part of pilot testing. One21seventy/ABCD tools and protocols are regularly reviewed by expert reference groups to ensure continued alignment with best practice standards. Audit data are collected manually by health centres and directly entered into the One21seventy online database system.

The criteria for inclusion in a Type 2 diabetes audit includes patients who: have a diagnosis of Type 2 diabetes; are aged 16 years or older; and have lived within community for six months or more in the last year. Where the eligible population is 30 or less, the audit protocol recommends that all eligible records at that health centre are audited. For 30 or more eligible clients, the protocol recommends and provides guidance for health centres to draw a sufficient number of records to achieve a precision of 90% or 95% confidence of the sample representing the population [15]. This sampling approach resulted in a total sample of 10,674 records available for analysis in this study.

Ethics approval was obtained from research ethics committees in each jurisdiction (Human Research Ethics Committee of the Northern Territory Department of Health and Menzies School of Health Research (HREC-EC00153); Central Australian Human Research Ethics Committee (HREC-12-53); New South Wales Greater Western Area Health Service Human Research Committee (HREC/11/GWAHS/23); Queensland Human Research Ethics Committee Darling Downs Health Services District (HREC/11/QTDD/47); South Australian Aboriginal Health Research Ethics Committee (04-10-319); Curtin University Human Research Ethics Committee (HR140/2008); Western Australian Country Health Services Research Ethics Committee (2011/27); Western Australia Aboriginal Health Information and Ethics Committee (111-8/05); University of Western Australia Human Research Ethics Committee (RA/4/1/5051)).

### Data analysis

To assess Type 2 diabetes service delivery, the study measured 15 items from best practice guidelines used across the states and territories. The service items included laboratory investigations (albumin creatinine ratio, estimated glomerular filtration rate, full lipid profile and glycosylated haemoglobin), physical checks (weight, waist circumference, body mass index, blood pressure, visual acuity, dilated eye check, foot check) and counselling for certain risk factors (nutrition, physical activity, tobacco and alcohol use). Process of care performance for each patient was calculated by determining the proportion of services received out of the 15 scheduled services. A mean adherence to delivery of Type 2 diabetes services in a given health centre represented an overall performance score for the health centre in a given audit cycle. Each aggregate score was converted into a binary outcome variable that categorised ‘higher’ performance as being within the top quartile of delivery across all health centres measured at baseline (greater than 76% service delivery). Health centre characteristics included length of participation in ABCD CQI, regularity of client attendance, size of service population, governance (community-controlled or government operated) and location based on the Australian Standard Geographical Classification (AGSC) system (very remote, remote or non-remote). For statistical analysis, rates of patient attendance were calculated based on the proportion of patients that did not attend in the previous six months prior to audit. A binary outcome variable was created that categorised a health centre as having ‘lower regular attendance’ if more than 3% of patients did not attend within the previous six months.

Patient level characteristics (sex, age, Indigenous status) were extracted from clinical records. Documented chronic health conditions (diabetes, hypertension, chronic heart disease and chronic kidney disease) were recorded as present or absent and co-morbidities were calculated by summing the recorded presence of each condition. Similarly, documented complications (retinopathy, neuropathy, foot ulcers and amputations) were recorded as present or absent and number of complications was calculated per individual.

Multilevel mixed effects logistic regression models were used to quantify the variation in Type 2 diabetes service delivery attributable to health centre or patient level factors, allowing for the hierarchical structure of the data (patients nested within health centres nested within jurisdictions). Crude odds ratios were calculated to measure the unadjusted association between the outcome and predictor variables. Non-significant variables were excluded from further analysis. Potential interactions were checked for significance. We adopted a stepwise modelling strategy starting with Model A that included the audit year variable only to test the influence of jurisdictions and health centres on Type 2 diabetes processes over time. Significant health centre (Model B) and then patient level variables (Model C) from the unadjusted analysis were introduced into the empty model. Predicted means and 95% confidence intervals were obtained from the regression analyses to rank jurisdictions and health centres according to their probability of providing a higher proportion of services compared to the overall mean probability (log odds scale).

The reduction in variance due to the stepwise introduction of the different variables in the models was determined by the proportional change in variance (PCV) at different levels. The PCV provides an estimate of the extent to which health centre or patient level factors may explain individual differences in propensity for better health care delivery [16]. Median odds ratios (MORs) were calculated to help interpret variance in the odds ratio scale. The MOR is the increased (median) probability of receiving ‘a high proportion of’ recommended care processes if a patient was to change health centre or jurisdiction [16,17]. If the MOR was equal to 1, there would be no difference between jurisdictions/health centres. If there were important differences between jurisdictions/health centres, the MOR would be large. The accuracy of the variance estimates was evaluated by their standard error (SE). A p-value ≥0.05 was considered non-significant. Statistical analyses were conducted with STATA software, V.13.

## Results

Of the 132 participating health centres, 79% were located in very remote or remote areas, 44% had a service population of 500 or fewer, and 73% were government operated (Table 2). In all jurisdictions, on average, approximately 90% of clients audited attended the health centre within the previous six months. By location, there was a higher mean percentage of clients not attending within the previous six months in non-remote areas (25%) compared to remote (11%) and very remote areas (5%; Table 3). The mean age of patients was 52 years and 57% were female. Over 90% of records from the Northern Territory, Western Australia and South Australia were for Aboriginal or Torres Strait Islander people, compared to 69% in Queensland and 55% in Far West New South Wales health centres. Around 71% of patients had co-morbidities and 14% had one or more diabetes complications. These occurred more commonly in older patients (Table 4).

**Table 2:** **Characteristics of health centres and patients with Type 2 diabetes by jurisdiction (number & (%))**

		**Far West New South Wales**	**Northern Territory**	**Queensland**	**South Australia**	**Western Australia**	**Total**
**Total number health centres**		**6**	**62**	**44**	**6**	**14**	**132**
**Location**	Very remote	2 (33)	51 (82)	29 (66)	2 (33)	5 (36)	89 (67)
	Remote	1 (17)	9 (15)	4 (9)	0	2 (14)	16 (12)
	Non-remote	3 (50)	2 (3)	11 (25)	4 (67)	7 (50)	27 (20)
**Governance**	Government	0	44 (71)	44 (100)	3 (50)	6 (43)	97 (73)
	Community-controlled	6 (100)	18 (29)	0	3 (50)	8 (57)	35 (27)
**Service Population**	≤500	2 (33)	35 (56)	18 (41)	1 (17)	2 (14)	58 (44)
	501-999	1 (17)	12 (19)	11 (25)	2 (33)	0	26 (20)
	≥1000	3 (50)	15 (24)	15 (34)	3 (50)	12 (86)	48 (36)
**Mean % (and range) clients that did not attend in last 6 months**		21 (3–47)	4 (0–22)	12 (0–45)	25 (12–49)	17 (0–50)	10 (0–50)
**Duration of participation in ABCD CQI**	<1 year	0	14 (23)	12 (27)	2 (33)	4 (29)	32 (24)
	1-2 years	0	26 (42)	19 (43)	4 (67)	6 (43)	55 (42)
	>3 years	6 (100)	22 (35)	13 (30)	0	4 (29)	45 (34)
**Number of patient records audited**		**936**	**4849**	**3407**	**333**	**1149**	**10674**
**Age (years) mean & (range)**		57 (19–94)	50 (15–91)	54 (15–97)	52 (16–86)	52 (17–88)	52 (15–97)
**Gender**	Male	441 (47)	1925 (40)	1587 (47)	136 (41)	472 (41)	4561 (43)
	Female	495 (53)	2924 (60)	1820 (53)	197 (59)	677 (59)	6113 (57)
**Indigenous status**	Indigenous	514 (55)	4684 (97)	2347 (69)	313 (94)	1071 (93)	8929 (84)
	Non-indigenous	384 (41)	127 (3)	591 (17)	20 (6)	56 (5)	1178 (11)
	Not recorded	38 (4)	38 (1)	469 (14)	0	22 (2)	567 (5)
**Two or more co-morbidities**		770 (82)	3570 (74)	2326 (68)	210 (63)	677 (59)	7553 (71)
**Complications**	No complications	781 (83)	4185 (86)	2832 (83)	302 (91)	1000 (87)	9100 (85)
	1-2 complications	148 (16)	613 (13)	510 (15)	27 (8)	134 (12)	1432 (13)
	>2 complications	7 (1)	51 (1)	65 (2)	4 (1)	15 (1)	142 (1)

**Table 3:** **Percentage of clients that did not attend the health centre within the last six months by location**

	**Number of health centres**	**Percent clients that did not attend in the last six months**			
**Location**		**Mean**	**SE**	**Min**	**Max**
Non-remote	27	25	2.76	2	50
Remote	16	11	2.30	3	36
Very Remote	89	5	0.60	0	27

**Table 4:** **Clients with co-morbidities and disease complications by age group**

	**Number of chronic conditions documented**		**Number of disease complications**			
**Age (years)**	**One condition**	**≥2 conditions**	**None**	**1-2**	**>2**	**Total**
	**n (%)**	**n (%)**	**n (%)**	**n (%)**	**n (%)**	
**15 to <25**	115 (65)	63 (35)	168 (94)	7 (4)	3 (2)	178
**25 to <40**	716 (39)	1114 (61)	1693 (93)	110 (6)	27 (1)	1830
**40 to <55**	1246 (29)	3035 (70)	3680 (85)	449 (10)	152 (4)	4281
**> = 55**	1044 (24)	3341 (76)	3559 (81)	602 (14)	224 (5)	4385
**Total**	3121 (29)	7553 (71)	9100 (85)	1168 (11)	406 (4)	10674

Of all health services participating in the study around one third (34%) completed three or more cycles, 42% completed one to two cycles and 24% completed baseline audit only. This does not reflect attrition of health services, as some services commenced participation at differing time periods (Table 5). Of those health centres only completing baseline audits, 75% commenced ABCD CQI participation in 2011 or 2012 (Table 1).

**Table 5:** **Duration of CQI participation by health centre characteristic**

		**Completed Baseline only**	**Completed 1–2 cycles**	**Completed > =3 cycles**	**Total**
		**n (%)**	**n (%)**	**n (%)**	**n (%)**
**Remoteness**	**Non-Remote**	6 (19)	16 (29)	5 (11)	27 (20)
	**Remote**	5 (16)	5 (9)	6 (13)	16 (12)
	**Very Remote**	21 (66)	34 (62)	34 (76)	89 (67)
	**Total**	32 (100)	55 (100)	45 (100)	132 (100)
**Governance**	**Community-controlled**	7 (22)	7 (13)	21 (47)	35 (27)
	**Government**	25 (78)	48 (87)	24 (53)	97 (73)
	**Total**	32 (100)	55 (100)	45 (100)	132 (100)
**Population**	**<=500**	16 (50)	28 (51)	14 (31)	58 (44)
	**501-999**	2 (6)	11 (20)	13 (29)	26 (20)
	**> = 1000**	14 (44)	16 (29)	18 (40)	48 (36)
	**Total**	32 (100)	55 (100)	45 (100)	132 (100)

The distribution of remoteness of centres did not differ markedly by duration of participation in CQI, for example, 66%, 62% and 76% of health centres completing baseline, 1–2 audit cycles and 3 or more audit cycles respectively were from very remote areas. The distribution of health centres by governance showed that 47% of health centres that completed 3 or more cycles were community-controlled organisations and there was also an equal distribution of health centres by service population size that completed 3 or more cycles (Table 5).

**Figure 1:**
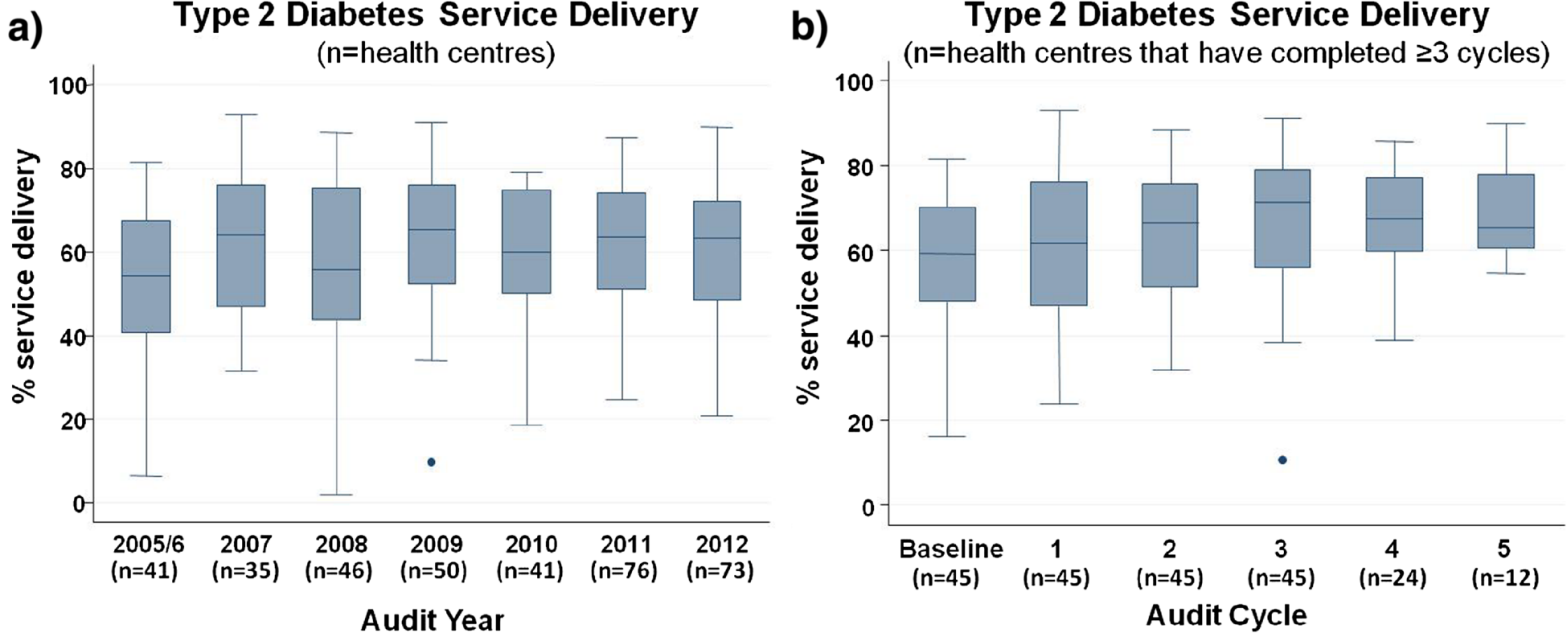
**Type 2 diabetes service delivery over time.** Mean percent Type 2 diabetes service delivery by **a)** all health centres over audit years (n = 132) and **b)** health centres that have participated in CQI for 3 or more years (n = 45) over successive audit cycles.

Wide variation in delivery of Type 2 diabetes services was evident across all health centres within an audit year and over time (Figure 1a). However, for those health centres that completed at least three audit cycles (n = 45) there was a trend of decreasing variation across successive cycles of CQI (Figure 1b). There were large improvements in service delivery in lower performing health centres from less than 20% in early cycles to 40-50% in later cycles (Figure 1b).

In explaining this variation, the unadjusted logistic regression analysis showed that duration of participation in the ABCD CQI program, remoteness, regularity of patient attendance, patient age, level of co-morbidity and number of disease complications, were significantly associated with improved Type 2 diabetes service delivery (Table 6). Remoteness of health centres showed the strongest association with very remote and remote centres having 4.57 (95% CI 2.61-8.01) and 2.55 (95% CI 1.22-5.35) times the odds of being in the top quartile of Type 2 diabetes service delivery compared to non-remote centres. We tested the interaction between remoteness and duration of CQI participation and found significant improvement in service delivery with length of participation irrespective of health centre location. There was, however, a gradient of increasing odds of improvement from non-remote, to remote and very remote centres. The odds of receiving top quartile service delivery also increased with increasing age, co-morbidity and disease severity. Considering the likelihood of a correlation between patient age and disease status, we tested and found a significant interaction between these variables. All interaction terms were incorporated into the final adjusted model as shown in Table 7.

**Table 6:** **Unadjusted multilevel regression analysis of health centre and patient level factors on delivery of guideline scheduled Type 2 diabetes services (n = 10674 clients; 132 health centres)**

***Outcome is >76% service delivery***		**Unadjusted**		
**Predictors**		**OR**	**95% CI**	**p-values**
Audit Year	2005/6	1.00	(reference)	
	2007	1.62	(1.32-1.98)	<0.0001
	2008	1.90	(1.57-2.32)	<0.0001
	2009	2.41	(1.99-2.93)	<0.0001
	2010	2.23	(1.80-2.76)	<0.0001
	2011	2.40	(1.97-2.93)	<0.0001
	2012	2.89	(2.37-3.53)	<0.0001
***Health Centre Characteristics***				
Location	Non-remote	1.00	(reference)	
	Remote	2.55	(1.22-5.35)	0.013
	Very Remote	4.57	(2.61-8.01)	<0.0001
Governance	Government operated	1.00	(reference)	
	Community-controlled	1.09	(0.64-1.87)	0.75
Service population	≤500	1.00	(reference)	
	>500- < 1000	1.38	(0.81-2.35)	0.24
	≥1000	0.80	(0.49-1.30)	0.37
Patient attendance in last 6 months	Lower attendance	1.00	(reference)	
	Higher attendance	1.57	(1.38-1.79)	<0.0001
Duration of CQI participation	Baseline	1.00	(reference)	
	1-2 cycles	1.86	(1.66-2.09)	<0.0001
	≥3 cycles	2.33	(2.03-2.68)	<0.0001
***Patient Characteristics***				
Sex	Male	1.00	(reference)	
	Female	1.06	(0.96-1.16)	0.24
Age (years)	≥15- < 25	1.00	(reference)	
	≥25- < 40	1.60	(1.09-2.35)	0.017
	≥40- < 55	2.16	(1.48-3.15)	<0.0001
	≥55	2.61	(1.79-3.81)	<0.0001
Indigenous status	Non-Indigenous	1.00	(reference)	
	Indigenous	1.03	(0.84-1.26)	0.80
Comorbidity	One condition	1.00	(reference)	
	≥2 conditions	1.54	(1.38-1.71)	<0.0001
Complications	None	1.00	(reference)	
	1-2 complications	1.44	(1.27-1.64)	<0.0001
	>2 complications	1.77	(1.20-2.60)	0.004

**Table 7:** **Adjusted multilevel regression analysis of health centre and patient level factors on delivery of guideline scheduled Type 2 diabetes services (n = 10,674 clients; 132 health centres)**

***Outcome is >76% service delivery***		**Empty Model A**		**Model B**		**Model C**	
**Predictors**	**Fixed effects**	**OR**	**95% CI**	**OR**	**95% CI**	**OR**	**95% CI**
Audit Year	2005/2006	1.00	(reference)	1.00	(reference)	1.00	(reference)
	2007	1.62	(1.32-1.98)***	1.14	(0.91-1.44)	1.20	(0.95-1.51)
	2008	1.90	(1.57-2.32)***	1.21	(0.95-1.54)	1.30	(1.02-1.67)*
	2009	2.41	(1.99-2.93)***	1.35	(1.03-1.77)*	1.40	(1.07-1.83)*
	2010	2.23	(1.80-2.76)***	1.16	(0.86-1.57)	1.17	(0.86-1.59)
	2011	2.40	(1.97-2.93)***	1.25	(0.92-1.71)	1.24	(0.91-1.69)
	2012	2.89	(2.37-3.53)***	1.18	(0.82-1.68)	1.17	(0.82-1.67)
***Health Centre Characteristics***							
Location X duration of CQI participation							
Non-remote:	Baseline			1.00	(reference)	1.00	(reference)
	1-2 cycles			1.53	(1.10-2.11)*	1.47	(1.06-2.04)*
	≥3 cycles			1.62	(1.02-2.55)*	1.55	(0.98-2.45)
Remote:	Baseline			1.00	(reference)	1.00	(reference)
	1-2 cycles			2.92	(1.36-6.24)**	2.91	(1.36-6.22)**
	≥3 cycles			3.14	(1.37-7.18)**	3.29	(1.44-7.54)**
Very Remote:	Baseline			1.00	(reference)	1.00	(reference)
	1-2 cycles			4.03	(2.27-7.16)***	4.31	(2.43-7.67)***
	≥3 cycles			4.72	(2.47-9.02)***	5.05	(2.63-9.67)***
Patient attendance in last 6 months	Lower attendance			1.00	(reference)	1.00	(reference)
	Higher attendance			1.40	(1.22-1.60)***	1.40	(1.22-1.61)***
***Patient Characteristics*** **(Age X disease status)**							
≥15- < 25 years: Comorbidity	One condition					1.00	(reference)
	2 or more conditions					1.83	(0.85-3.94)
≥15- < 25 years: Complications	None					1.00	(reference)
	1-2 complications					0.87	(0.13-5.68)
	>2 complications					5.82	(0.45-74.5)
≥25- < 40 years: Comorbidity	One condition					1.00	(reference)
	2 or more conditions					1.38	(1.10-1.73)**
≥25- < 40 years: Complications	None					1.00	(reference)
	1-2 complications					2.40	(1.53-3.77)***
	>2 complications					1.73	(0.72-4.13)
≥40- < 55 years: Comorbidity	One condition					1.00	(reference)
	2 or more conditions					1.43	(1.20-1.69)***
≥40- < 55 years: Complications	None					1.00	(reference)
	1-2 complications					1.32	(1.05-1.65)*
	>2 complications					1.17	(0.80-1.70)
≥55 years: Comorbidity	One condition					1.00	(reference)
	2 or more conditions					1.26	(1.05-1.52)*
≥55 years: Complications	None					1.00	(reference)
	1-2 complications					1.29	(1.05-1.58)*
	>2 complications					1.45	(1.04-2.01)*
**Random effects (intercepts)**							
State (variance (SE))		1.41	(0.99)	0.72	(0.53)	0.69	(0.51)
	**MOR**_**STATE**_	3.10		2.24		2.21	
*PCV (% explained variance)*				*49%*		*51%*	
Health Centre (variance (SE))		1.21	(0.19)	0.93	(0.15)	0.94	(0.15)
	**MOR**_**HC**_	2.86		2.51		2.52	
*PCV (% explained variance)*				*23%*		*23%*	
State & Health Centre (variance)		2.62		1.65		1.63	
	**MOR**_**STATE-HC**_	4.68		3.41		3.38	
*PCV (% explained variance)*				*37%*		*38%*	
Patient (variance (SE))		0.13	(0.072)	0.07	(0.03)	0.02	(0.012)
*PCV (% explained variance)*				*44%*		*81%*	

*p < 0.05; **p < 0.01; ***p < 0.0001.

MOR (Median odds ratio): odds of receiving ‘top quartile service delivery’ if a patient was to change health centre or jurisdiction [17].

PCV (Proportional change in variance): percent variation explained in odds for better health care delivery by introduction of health centre or patient level factors [16].

There was a significant effect of time, with a steady increase in the odds of health centres being within the top quartile of Type 2 diabetes service delivery from baseline to 2012. Health centres had approximately three times greater odds of top quartile performance in 2012 compared to 2005/2006 (95% CI 2.37-3.53) (Table 7, Model A). The effect of time diminished after adjusting for health centre and patient level factors (Table 7, Models B and C).

The PCV in Model B (Table 7) shows that the addition of health centre factors explained 37% of the variation across jurisdictions and health centres. The longer the duration of participation in the ABCD CQI program, the greater the odds of being within the top quartile of service delivery, particularly for health centres in remote and very remote locations (remote:1–2 cycles OR = 2.92, 95% CI 1.36-6.24; ≥3 cycles OR = 3.14, 95% CI 1.37-7.18; very remote: 1–2 cycles OR = 4.03, 95% CI 2.27-7.16; ≥3 cycles OR = 4.72, 95% CI 2.47-9.02). Health centres with a higher proportion of patients that attended within the previous six months also had better odds of being within the top quartile (OR = 1.40; 95% CI 1.22-1.60). The addition of patient level factors in Model C had a modest effect, accounting for a further 1% of the variation in service delivery across health centres and jurisdictions. In most age groups, patients with co-morbidities and disease complications had greater odds of receiving higher level care than those without these conditions.

**Figure 2:**
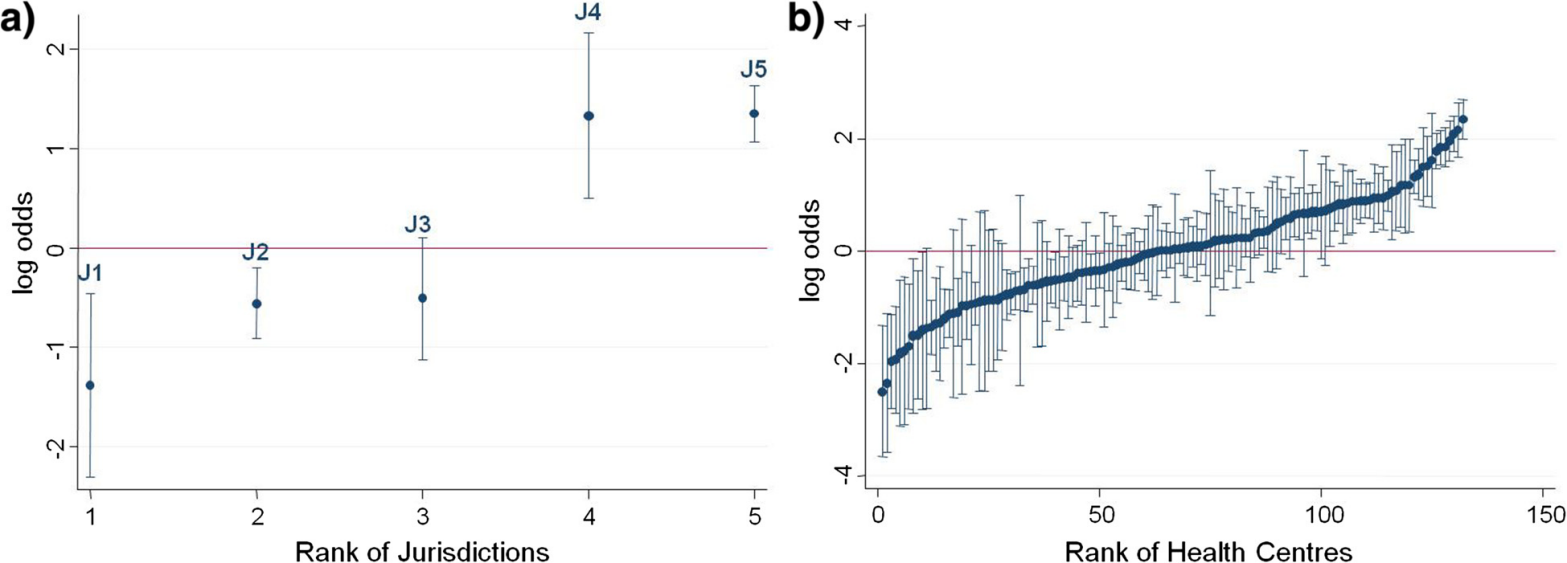
**Variation in adherence to guidelines on best practice Type 2 diabetes service delivery before adjustment.** Ranking of **a)** jurisdictions and **b)** health centres according to their average delivery of Type 2 diabetes services relative to the overall average delivery as predicted from the regression Model A (adjusting for audit year only). Uncertainty around this estimate is illustrated by 95% confidence intervals.

**Figure 3:**
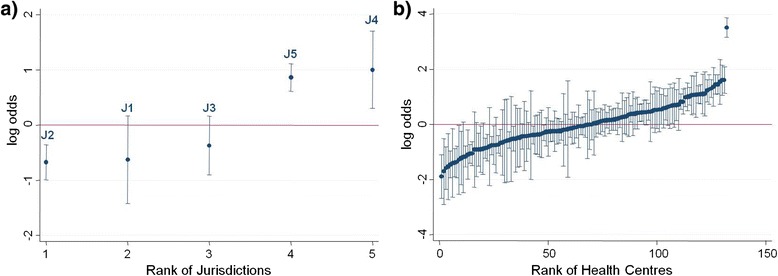
**Variation in adherence to guidelines on best practice Type 2 diabetes service delivery after adjustment.** Ranking of **a)** jurisdictions and **b)** health centres according to their average delivery of Type 2 diabetes services relative to the overall average delivery as predicted from the regression Model C (adjusting for health centre and patient level factors). Uncertainty around this estimate is illustrated by 95% confidence intervals.

The variation in the odds of top quartile delivery was reduced between jurisdictions and health centres after accounting for health centre and patient variables from MOR_STATE-HC_ = 4.68 (Model A; Table 7) to MOR_STATE-HC_ = 3.38 (Model C; Table 7). Changes in predicted odds for top quartile service delivery after adjusting for time and health centre and patient factors are shown in Figures 2 and 3 respectively. The highest performing outlier after adjusting for health centre and patient characteristics (Figure 3b) is a centre from a non-remote area that had completed one ABCD CQI Type 2 diabetes audit cycle since it joined in 2010. This health centre is renowned for its clinical leadership and model of care suited to the local Aboriginal and Torres Strait Islander population; factors which are not accounted for in this current analysis.

## Discussion

There were improvements over time in the level of Type 2 diabetes care across the diverse range of Aboriginal and Torres Strait Islander primary health centres participating in a wide-scale CQI project. The main factors associated with improvement were duration of participation in CQI, the location of the health centre, regularity of patient attendance and patient age, co-morbidity and disease severity.

A significant proportion of the variation across jurisdictions and centres was explained by health centre factors within the model (37%). There is an independent relationship between the duration of participation in ABCD CQI and improved documented adherence to best practice guidelines, with centres that participated for 3 or more cycles having between 1.5 to 5 times the odds of top quartile service delivery to patients depending on location (Model C; Table 7). This finding indicates that health centres in varying geographical contexts appear to obtain benefits from sustained CQI participation. Long term commitment to CQI was also associated with improvements in health centres at the lower range of performance, with an increase in the lowest levels of adherence to best practice guidelines from less than 20% to approximately 40% over three audit cycles (Figure 1b).

Health centres located in very remote (ASGC-RA 5) and remote areas (ASGC-RA 4) had increased odds of greater improvement in care processes over audit cycles compared to non-remote health centres. The ASGC-RA classification system is based on physical distance of the community to the nearest urban area and reflects access to goods and services [18]. The greater odds of a higher level of service delivery in these areas may reflect less complex service environments where the centre may be the single primary health care provider and some distance away from other providers. The availability of multiple service providers in non-remote locations provides a challenge for primary health care centres in coordinating and monitoring comprehensive delivery of care to patients with chronic disease. Yet, the quality of care shown by remote health centres has occurred in service environments characterised by high staff turnover, high use of locums and limited access to specialist services. Higher levels of service delivery in these areas may be due to the implementation of specific models of primary health care delivery (for example, hub and spoke and outreach services) designed to overcome barriers of remote geography, dispersed populations and to improve coordination of care [19]. CQI may have contributed in part to the development of these models of care as indicated in a recent evaluation of the CQI Strategy in the Northern Territory that suggested CQI processes led to positive changes in health centre practices and an increase in service delivery outputs [20].

Best practice Type 2 diabetes care dictates that patients receive certain services at regular intervals. It is unsurprising that health centres with a higher percentage of patients (≥3%) not attending in the previous six months had lower odds of providing best practice care. While non-remote health centres had on average 25% clients not attending in the previous six months compared to 11% and 5% of patients in remote and very remote areas respectively (Table 3), there was no significant statistical interaction between regularity of patient attendance and remoteness of health centres with respect to delivery of Type 2 diabetes care.

The addition of patient-level characteristics into the model explained only a small component of the variation between health centres and jurisdictions (1%). There was an increasing trend of co-morbidity and disease severity with age (Table 4) and those patients who were older, had co-morbidities and/or complications were more likely to have greater odds of receiving top quartile service delivery. Within most age groups, patients with co-morbidities and complications had greater odds of receiving more services compared to those without. This finding may reflect more opportunities for providing care or greater emphasis on managing high risk patients. Higher levels of care for high need patients along with general improvements in overall standards of care should contribute to a reduction in the Type 2 diabetes disease burden on health care systems and improved population health outcomes.

There was a consistent trend of increasing odds of top quartile service delivery over audit years compared to the baseline year 2005/6 apart from a flattening out period in 2010 (Table 7, Model A). The 2010–2011 period marked a new expansion phase for the ABCD project with new centres joining and the commencement of a nation-wide CQI service support agency (One21seventy) designed to assist with implementation of the ABCD CQI tools and processes. Also in 2010, two jurisdictions implemented a network of regional CQI facilitators to support health centres with their CQI activities. The number of health centres participating and the number of audits undertaken, including Type 2 diabetes, increased substantially from 2011 once the new support infrastructure was in place. Jurisdictional differences in the odds of health centres being within the top quartile of service delivery may be attributed to the variable state-wide level support systems as well as the staggered commencement of participation in the ABCD project. Employment of regional positions that supported collaboration and information exchange across health centres was a key explanatory factor for improved performance at health centre level over time [21]. The top two ranked jurisdictions with respect to odds of top quartile delivery, before and after adjusting for health centre and patient factors (Figures 2a and 3a), are those that participated for more years in ABCD and/or have a regional CQI facilitator network. Similar factors have been attributed to the success of a long term diabetes quality of care project in a remote community of Western Australia [22]. Learnings from jurisdictions where there is well established macro-level CQI infrastructure can support the development of similar structures in other jurisdictions [23].

The investigation of patient, health centre and jurisdictional level factors that underlie variation in adherence to best practice Type 2 diabetes care has been made possible by the availability of the extensive CQI audit dataset from health centres participating in the ABCD National Research Partnership. The voluntary nature of participation in this study by health centres, however, limits the generalisability of study findings. In addition, as data are collected from client records, service delivery may be underestimated due to poor documentation. Although a strength of this study design is the ability to correct for a range of known confounding factors, it is also recognised that the associations identified in this study, may be due, in part, to other unmeasured confounders. For example, there may be factors associated with longer term participation in CQI that may be responsible for the demonstrated improvements in adherence to best practice guidelines, such as well-functioning community health centres with good leadership and workforce stability that are both more likely to maintain CQI processes, and to achieve better results in diabetes management. The exemplary centre in Figure 3b is a case in point where their level of service delivery is amongst the best despite relatively limited participation in ABCD CQI to date. As mentioned, the health centre is renowned for its clinical leadership and model of care built around its own local CQI processes. To elucidate other potentially confounding factors enabling quality improvement, further quantitative and qualitative studies are planned to examine micro-, meso- and macro-level factors that have facilitated sustained improvement in high performing health centres [24]. It is important to note that it is through participation in the CQI initiative that data are available for the sort of analysis presented in this study. There are currently no other sources of comparable data in Australian primary care that would enable establishment of a comparison group not participating in the CQI program.

## Conclusions

The findings of this study suggest that at the health centre level, the quality of Type 2 diabetes care for Aboriginal and Torres Strait Islander communities is associated with long term commitment to a CQI program. Sustained participation has particularly benefitted remote and very remote health centres and health centres performing at the lower range of service provision. Our findings suggest that improving regularity of patient attendance, better coordination and documentation of care within non-remote multiple service provider environments and sharing lessons from the successes of care coordination in remote areas has the potential to improve care delivery across the spectrum of health centres. Further understanding of system wide factors that underpin jurisdictional variation in delivery of care will assist health managers and policy makers to understand and develop macro-level strategies to improve quality of Type 2 diabetes care in Aboriginal and Torres Strait Islander communities.

## Abbreviations

ABCD: Audit and Best Practice for Chronic Disease
ASGC: Australian Standard Geographical Classification
CQI: Continuous quality improvement
MOR: Median odds ratio
OR: Odds ratio
PCV: Proportional change in variance
PHC: Primary health care
SE: Standard error
